# Dynamic footprints of α-synucleinopathic mice recorded by CatWalk gait analysis

**DOI:** 10.1016/j.dib.2017.12.067

**Published:** 2018-01-03

**Authors:** Ivanna K. Timotius, Fabio Canneva, Georgia Minakaki, Cristian Pasluosta, Sandra Moceri, Nicolas Casadei, Olaf Riess, Jürgen Winkler, Jochen Klucken, Stephan von Hörsten, Bjoern Eskofier

**Affiliations:** a Dept. of Computer Science, Faculty of Engineering, Friedrich-Alexander-University Erlangen-Nürnberg (FAU), Germany; b Dept. Experimental Therapy, University Hospital Erlangen (UKEr) and Preclinical Experimental Animal Center, Friedrich-Alexander-University Erlangen-Nürnberg (FAU), Germany; c Dept. of Molecular Neurology, University Hospital Erlangen, University of Erlangen-Nürnberg (FAU), Germany; d Dept. of Electronics Engineering, Satya Wacana Christian University, Salatiga, Indonesia; e Dept. of Microsystems Engineering, University of Freiburg, Germany; f Institute of Medical Genetics and Applied Genomics, University of Tübingen, Germany

## Abstract

Characterizing gait is important in the study of movement disorders, also in clinical mouse models. Gait data are therefore necessary for the development of gait analysis methods and the study of diseases. This article presents gait data of two α-synucleinopathic transgenic mouse models and their non-transgenic littermate, backcrossed into the C57BL/6N genetic background. The animal gait was recorded using CatWalk system, which provides the information for each run about the paw positions, paw print sizes, and paw intensities as a function of time or video frame. A total of 90 run data files are provided in this article.

**Specifications Table**

**Table:** 

Subject area	*Biology, Behavior Science, Neuroscience, Computer Science*
More specific subject area	*Gait analysis in mice*
Type of data	*Spreadsheet files (.xlsx)*
How data was acquired	*CatWalk locomotion test*
Data format	*Raw*
Experimental factors	*The run data were recorded from voluntary walking with minimum external disturbances*
Experimental features	*Paw positions, paw print sizes, and paw intensities of α-synucleinopathic mice and their non-transgenic littermates were recorded using CatWalk system*
Data source location	*Erlangen, Germany. (49.59°N, 11.01°E)*
Data accessibility	*The data are with this article*
Related research article	*I.K. Timotius, F. Canneva, G. Minakaki, C. Pasluosta, S. Moceri, N. Casadei, O. Riess, J. Winkler, J. Klucken, S. von Hörsten, B. Eskofier, Dynamic footprint based locomotion sway assessment in α-synucleinopathic mice using Fast Fourier Transform and Low Pass Filter, J. Neurosci. Methods. 296 (2018) 1-11.*

**Value of the Data**

• The data presented in this article are dynamic footprint data of α-synucleinopathic mice and their non-transgenic littermates as a function of time or video frame.
• Two different α-synuclein transgenic mouse models were included in the experiment.
• The data allows researchers to develop gait analysis methods based on the rodent dynamic footprints

## 1 Data

**Fig. 1:**
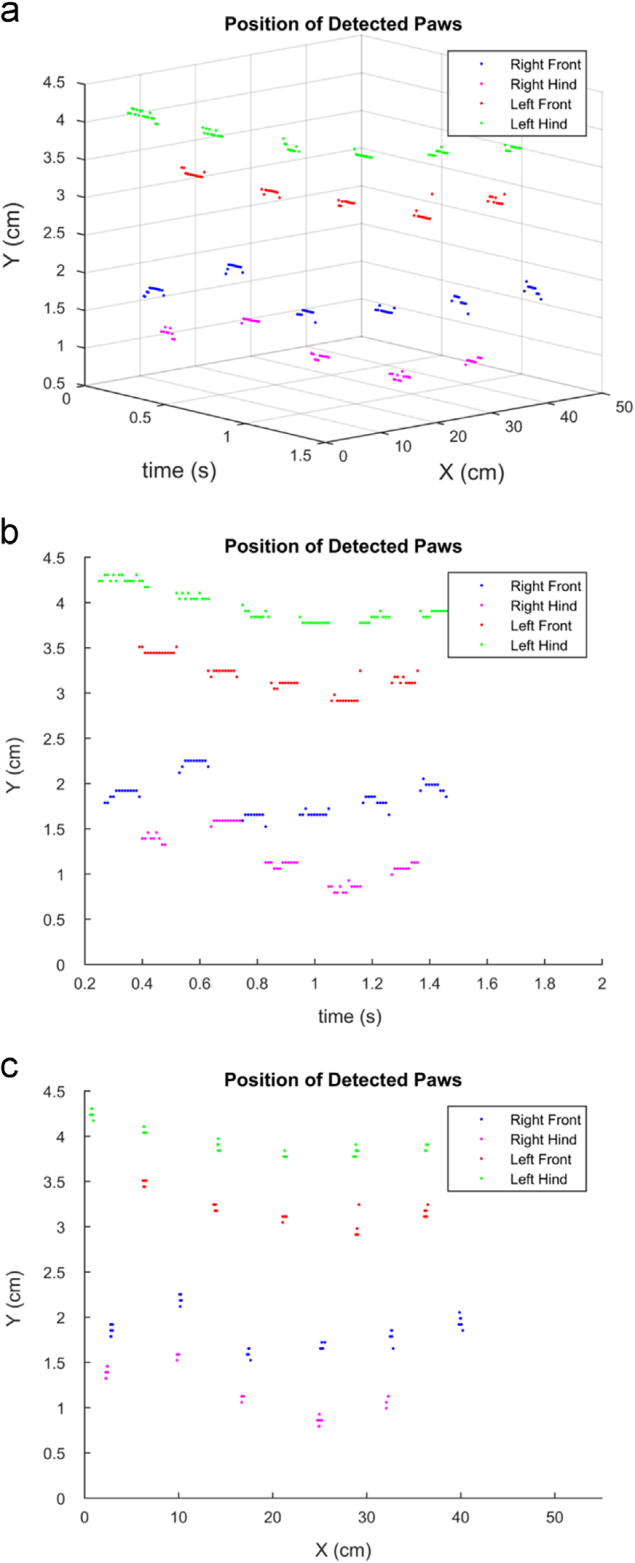
An example of the paw position plot generated from the data recorded from a wild-type mouse (a) as a function of the paw position in x-axis and time (b) as a function of time (c) as a function of the paw position in x-axis.

The data provided in this article are the raw locomotion data exported from CatWalk System [1]. The data have been used in developing the methods to assess of locomotion sway [2]. Laboratory wild-type mice and two different α-synuclein transgenic Parkinson's disease-relevant mouse models (α-syn_m_-ko and α-syn_m_-ko x α-syn_h_-tg) were used in the experiment. Each mouse runs several times on the walkway, and a data file was generated from each run. The information provided in each file are the frame numbers and their timestamps along with the information from each paw, namely, the paw positions in x- and y-axis (cm), print length (cm), print width (cm), print area (cm^2^), minimum intensity, maximum intensity, and the mean of intensity. Fig. 1 shows an example of the paw position plot.

## 2 Experimental design, materials, and methods

**Fig. 2:**
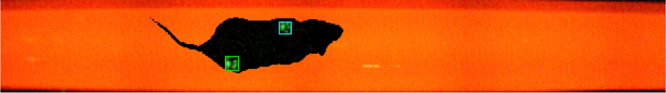
An example of an image captured from a video recorded from a wild-type mouse.

The footprints of mice were assessed with CatWalk system [1], [3], [4], which is a sensitive tool equipped with an enclosed walkway. Rodents could voluntarily walk on a glass plate in a darkened room. Green light is scattered on this glass plate at the points were their paw touch, which is captured by a video-camera underneath at a sampling rate of 100 Hz. An example of an image captured from the recorded video is shown in Fig. 2.

Seven-month-old male laboratory mice, backcrossed into the C57BL/6N genetic background, were used. Three different genotypes are included in the experiment:

a) 12 non-transgenic mice (wild-type or “WT” mice),
b) 13 mice bearing a knock-out for the endogenous murine aSyn (α-syn_m_-ko or “KO” mice, described by Abeliovich and colleagues [5]),
c) 8 mice double transgenic mice, which expressing exclusively the human aSyn, under the control of a bacterial artificial chromosome construct on the aSyn KO background (α-syn_m_-ko x α-syn_h_-tg or “huWT/KO” mice, described by Kohl and colleagues [6]).

The mouse weight is 31±2.47 g (mean±SD). No significant weight differences across the three genotypes are observable. The animals were maintained under standard laboratory conditions on a 12 h/12 h light/dark cycle, with lights on at 6:00 a.m. and lights off at 6:00 p.m., under specific-pathogen-free conditions. They were provided with food and water ad libitum. All procedures were in accordance with guidelines approved by the local Animal Welfare and Ethics committee of Bavaria, Germany (RegUFr#55.2-2532-2-218).

During the data acquisition, each mouse was placed on the walkway in a dark environment and could walk freely in both directions with a minimum level of external disturbing factors. The CatWalk software automatically recorded the video, where the mouse covered the whole distance of the walkway. Experimental sessions are typically last for 5–10 min. After a data acquisition each subject was returned to its own home-cage, in order to reduce habituation of the animals to the environment and the appearance of unwanted behaviors (e.g. sniffing, rearing and sitting).

From each mouse 2–4 compliant runs were recorded. The compliancy of a run is first determined automatically by the CatWalk system according to the run's duration and speed variation [1] to omit the runs with stopping or turning on the alley, then by an experienced observer to omit the unwanted behaviors. The total compliant runs recorded from WT, KO, and huWT/KO mice were respectively 34, 32, and 24. The paw positions were first automatically labeled by the CatWalk system, then revised by an experienced observer. The minimum detected print intensity was set at 0.12 or 0.16 and the camera gain was set at 30.19 or 29.56. The information about paw positions, print sizes and intensities are registered in the run data files, which are provided in this article.
